# Identification and Verification of Potential Hub Genes in Amphetamine-Type Stimulant (ATS) and Opioid Dependence by Bioinformatic Analysis

**DOI:** 10.3389/fgene.2022.837123

**Published:** 2022-03-30

**Authors:** Wei Zhang, Xiaodong Deng, Huan Liu, Jianlin Ke, Mingliang Xiang, Ying Ma, Lixia Zhang, Ming Yang, Yun Liu, Feijun Huang

**Affiliations:** ^1^ Department of Forensic Pathology, West China School of Basic Medical Science & Forensic Medicine, Sichuan University, Chengdu, China; ^2^ Department of Forensic Pathology, School of Basic Medical Science & Forensic Medicine, North Sichuan Medical College, Nanchong, China; ^3^ Department of Preventive Medicine, North Sichuan Medical College, Nanchong, China; ^4^ Department of Neurology, Affiliated Hospital of North Sichuan Medical College, Nanchong, China; ^5^ Department of Criminal Investigation, Nanchong Municipal Public Security Bureau, Nanchong, China; ^6^ Medical Imaging Key Laboratory of Sichuan Province, North Sichuan Medical College, Nanchong, China

**Keywords:** amphetamine-type stimulants (ATS), opioids, differentially expressed genes (DEGs), PI3K/Akt pathway, apoptosis, hub gene

## Abstract

**Objective**: Amphetamine-type stimulant (ATS) and opioid dependencies are chronic inflammatory diseases with similar symptoms and common genomics. However, their coexpressive genes have not been thoroughly investigated. We aimed to identify and verify the coexpressive hub genes and pathway involved in the pathogenesis of ATS and opioid dependencies.

**Methods**: The microarray of ATS- and opioid-treatment mouse models was obtained from the Gene Expression Omnibus database. GEO2R and Venn diagram were performed to identify differentially expressed genes (DEGs) and coexpressive DEGs (CDEGs). Functional annotation and protein–protein interaction network detected the potential functions. The hub genes were screened using the CytoHubba and MCODE plugin with different algorithms, and further validated by receiver operating characteristic analysis in the GSE15774 database. We also validated the hub genes mRNA levels in BV2 cells using qPCR.

**Result**: Forty-four CDEGs were identified between ATS and opioid databases, which were prominently enriched in the PI3K/Akt signaling pathway. The top 10 hub genes were mainly enriched in apoptotic process (CD44, Dusp1, Sgk1, and Hspa1b), neuron differentiation, migration, and proliferation (Nr4a2 and Ddit4), response to external stimulation (Fos and Cdkn1a), and transcriptional regulation (Nr4a2 and Npas4). Receiver operating characteristic (ROC) analysis found that six hub genes (Fos, Dusp1, Sgk1, Ddit4, Cdkn1a, and Nr4a2) have an area under the curve (AUC) of more than 0.70 in GSE15774. The mRNA levels of Fos, Dusp1, Sgk1, Ddit4, Cdkn1a, PI3K, and Akt in BV2 cells and GSE15774 with METH and heroin treatments were higher than those of controls. However, the Nr4a2 mRNA levels increased in BV2 cells and decreased in the bioinformatic analysis.

**Conclusions:** The identification of hub genes was associated with ATS and opioid dependencies, which were involved in apoptosis, neuron differentiation, migration, and proliferation. The PI3K/Akt signaling pathway might play a critical role in the pathogenesis of substance dependence.

## 1 Introduction

The amphetamine-type stimulants (ATS) and opioids are the major powerful and highly addictive drugs worldwide and nationwide, including 3,4-methylenedioxy-methamphetamine (MDMA), methamphetamine (METH), heroin, morphine, and opium (https://www.unodc.org/unodc/en/data-and analysis/wdr2021.html). Ellis et al. reported that the ATS- and opioid-exposure patients increased from 18.8% in 2011 to 34.2% in 2017 (Ellis et al., 2018). An acute dose of drug treatment led to neuronal death in the frontal cortex, striatum, and substantia nigra in animals, whereas repeated drug administration/chronic exposure led to neuronal loss in the hippocampus, frontal cortex, and striatum (Sabrini et al., 2020). Chronic and repeated administration could also cause tolerance and dependence, recurrent encephalopathy (e.g., neuronal degeneration and/or damage, neuroinflammation), and epigenetic modifications (e.g., histone modifications, DNA methylation, and noncoding RNAs) (Feng and Nestler 2013). Polysubstance dependence, especially opioid and ATS dependence, is evolving into an epidemic drug use pattern worldwide (Jones et al., 2019; Cicero et al., 2020). There are similar target genes (e.g., FAAH, BDNF, DRD4, and OPRM1) and mechanisms (e.g., DNA damage, apoptosis, neurotoxicity, neuroinflammation, and epigenetic modifications) between ATS and opioid dependence (Lopez-Moreno et al., 2012; Feng and Nestler 2013; Górska et al., 2014; Silva et al., 2014; Moratalla et al., 2017; Doris et al., 2019; Zhang et al., 2020; Lopez-Leon et al., 2021). However, the potential coexpressive genes and pathogenesis of ATS and opioid dependence have not been thoroughly investigated. The coexpressive genes were widely applied to discern candidate biomarkers and therapeutic targets for Alzheimer’s disease, schizophrenia, immune-mediated inflammatory diseases, and cancer (Zhu et al., 2016; van Dam et al., 2017; Kakati et al., 2019; Chen Y. et al., 2020; Wang et al., 2021). We hypothesized that coexpressive target genes and signaling pathways may provide further insight into the common pathophysiological process of ATS and opioid dependencies.

Following the development of bioinformatic technology, RNA sequencing (RNAseq) and high-throughput microarray had been widely used to explore and detect the biomarkers, functional annotation, and molecular mechanism of a variety of diseases *in vivo* and *in vitro* in the past decades (Kang et al., 2021; Sun et al., 2021; Xu et al., 2021). All the databases could be downloaded and reanalyzed freely. As the most economic and effective technique, bioinformatic analysis was performed to identify candidate hub genes. Previous microarrays mainly focused on a single drug, such as nicotine (Jung et al., 2016), alcohol (Smith et al., 2020), morphine (Skupio et al., 2017), heroin (Kuntz-Melcavage et al., 2009), MDMA (Eun et al., 2010), METH (Palmer et al., 2005), or cocaine (Renthal et al., 2009). They mainly focused on comparatively small samples, a single timepoint, or individual reward brain regions (Piechota et al., 2010; Piechota et al., 2012). Therefore, we conducted the integrated bioinformatic analysis with all conditions and timepoints in all GEO databases. Receiver operating characteristic (ROC) analysis and quantitative real-time PCR (qPCR) were performed to validate the hub genes and key factors of the signaling pathway between ATS and opioid treatments.

## 2 Materials and methods

### 2.1 Data collection

**FIGURE 1:**
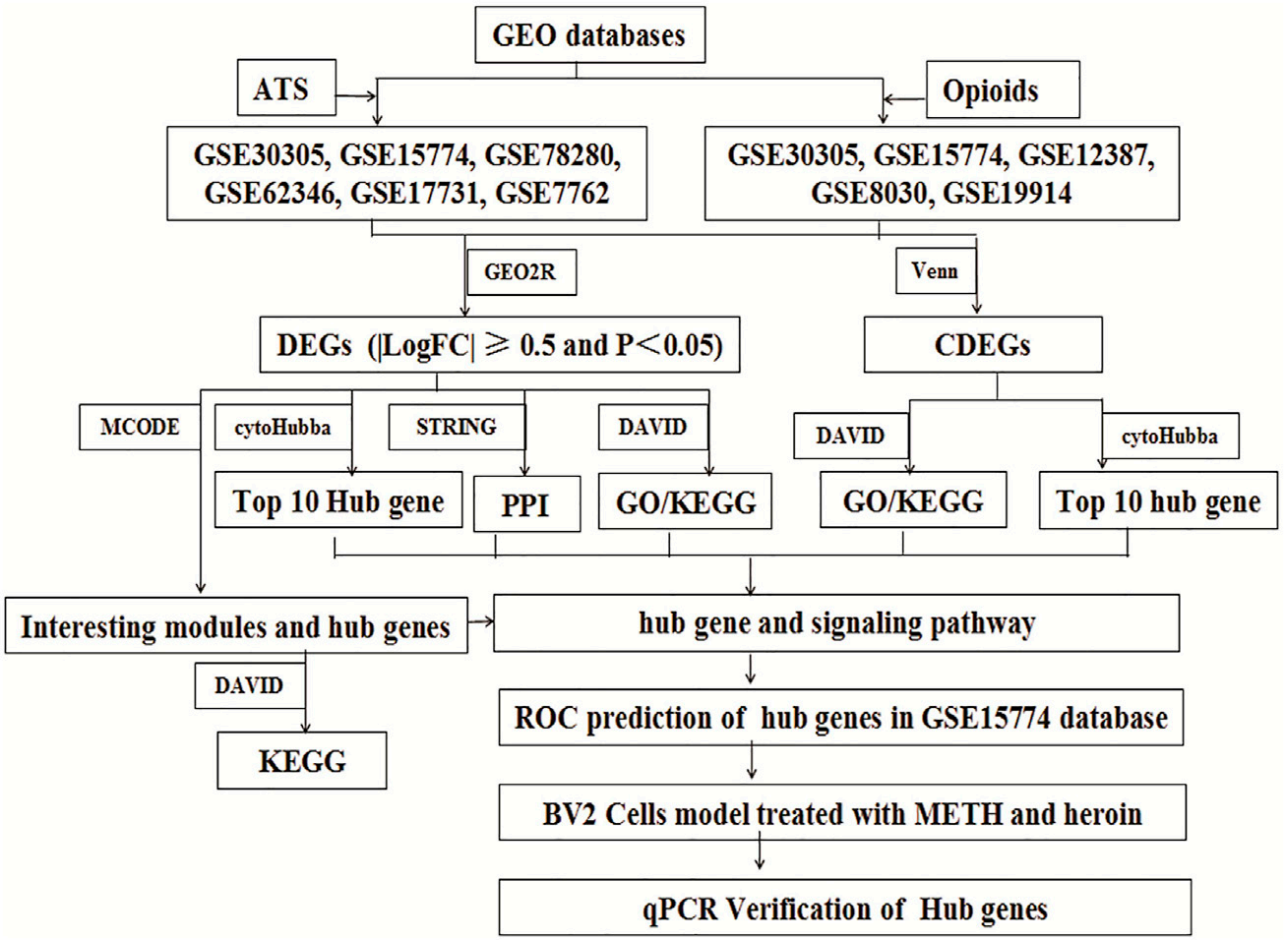
Flow diagram of the study design.

The study was approved by the Ethics Committee of Sichuan University. The microarrays were systematically extracted from the Gene Expression Omnibus (GEO) databases (http://www.ncbi.nlm.nih.gov/geo/) with several keywords: “heroin” or “morphine” or “opioids” or “opium,” “Amphetamine” or “methylamphetamine” or “METH” or “MA” or “3,4-methylenedioxymethamphetamine” (Barrett et al., 2010). The inclusion criteria include the following: 1) Databases were restricted in “expression profiling by microarrays.” 2) The organism was limited to “*Mus musculus*” brain. 3) The original microarrays should contain cases and controls. The exclusion criteria were the following: 1) The mice were intervened by other drugs. 2) Transgenic mice. 3) Other tissues. In addition, for overlapping databases, only the maximum samples were included. The workflow of the study is shown in Figure 1.

### 2.2 Differentially expressed gene analysis

GEO2R (https://www.ncbi.nlm.nih.gov/geo/geo2r) was utilized to screen and identify the DEGs from all the eligible databases, respectively (Davis and Meltzer 2007). If a gene had multiple probes on the same chip, the average value of all probes would be taken as the gene expression value. If the genes lacked probes, they were removed. The DEGs were defined with |log2 fold change (FC)| ≥ 0.5 and *p *<* *0.05 in the study. The coexpressive DEGs (CDEGs) were the overlapped DEGs of ATS and opioid treatment, which were screened by a Venn diagram (version 2.1.0, https://bioinfogp.cnb.csic.es/tools/venny/index.html).

### 2.3 Functional analysis and construction of protein–protein interaction network

Gene Ontology (GO) (Ashburner et al., 2000) and Kyoto Encyclopedia of Genes and Genomes (KEGG) (Kanehisa et al., 2019) pathway enrichment analyses of DEGs and CDEGs were applied to explore the biological function and signaling pathway by the online tool Database for Annotation, Visualization, and Integrated Discovery (DAVID, version 6.8 https://david.ncifcrf.gov/). A term with *p < 0*.*05* was identified as the critical threshold for significant enrichment.

Protein–protein interaction (PPI) network revealed the specific and nonspecific interactions of proteins by the Search Tool for the Retrieval of Interacting Genes database (STRING, https://string-db.org/) (Szklarczyk et al., 2019). The minimum required interaction score of more than 0.4 and *p*-value less than 0.05 was statistically significant (Fang et al., 2021; Zhou et al., 2021). The Cytoscape software (version 3.6.1) was applied to visualize the PPI network (Shannon et al., 2003). Besides, the Molecular Complex Detection (MCODE) plugin in the Cytoscape software was used to identify the interesting modules (selection criteria: degree cutoff = 2, K-core = 2, and node score cutoff = 0.2) (Bader and Hogue, 2003; Bandettini et al., 2012; Su et al., 2021).

### 2.4 Hub genes screened

Four most effective algorithms in the CytoHubba plugin in Cytoscape software were performed to identify hub genes, including the maximal clique centrality (MCC), density of maximum neighborhood component (DMNC), maximum neighborhood component (MNC), and degree. A high score indicated that the target was closely related to the disease and was a possible hub gene. The MCODE plugin in the Cytoscape software were also used to identify the hub genes by MCODE score (top three connective genes in interesting modules) (Chin et al., 2014; Nangraj et al., 2020; Qi et al., 2021).

### 2.5 Transcription factor target regulatory network

The TF-targeted top 20 hub genes of the PPI network were predicted with the iRegulon plugin in the Cytoscape software, which integrated information from the larger modify and track collections. We obtained the data predicted by the track discovery of existing regulatory databases, which included data validated by ChIP-seq, DHS-seq, or FAIRE-seq. TF-target pairs with normalized enrichment score (NES) >4 were chosen (Janky et al., 2014).

### 2.6 Quantitative real-time PCR verified hub genes

**FIGURE 2:**
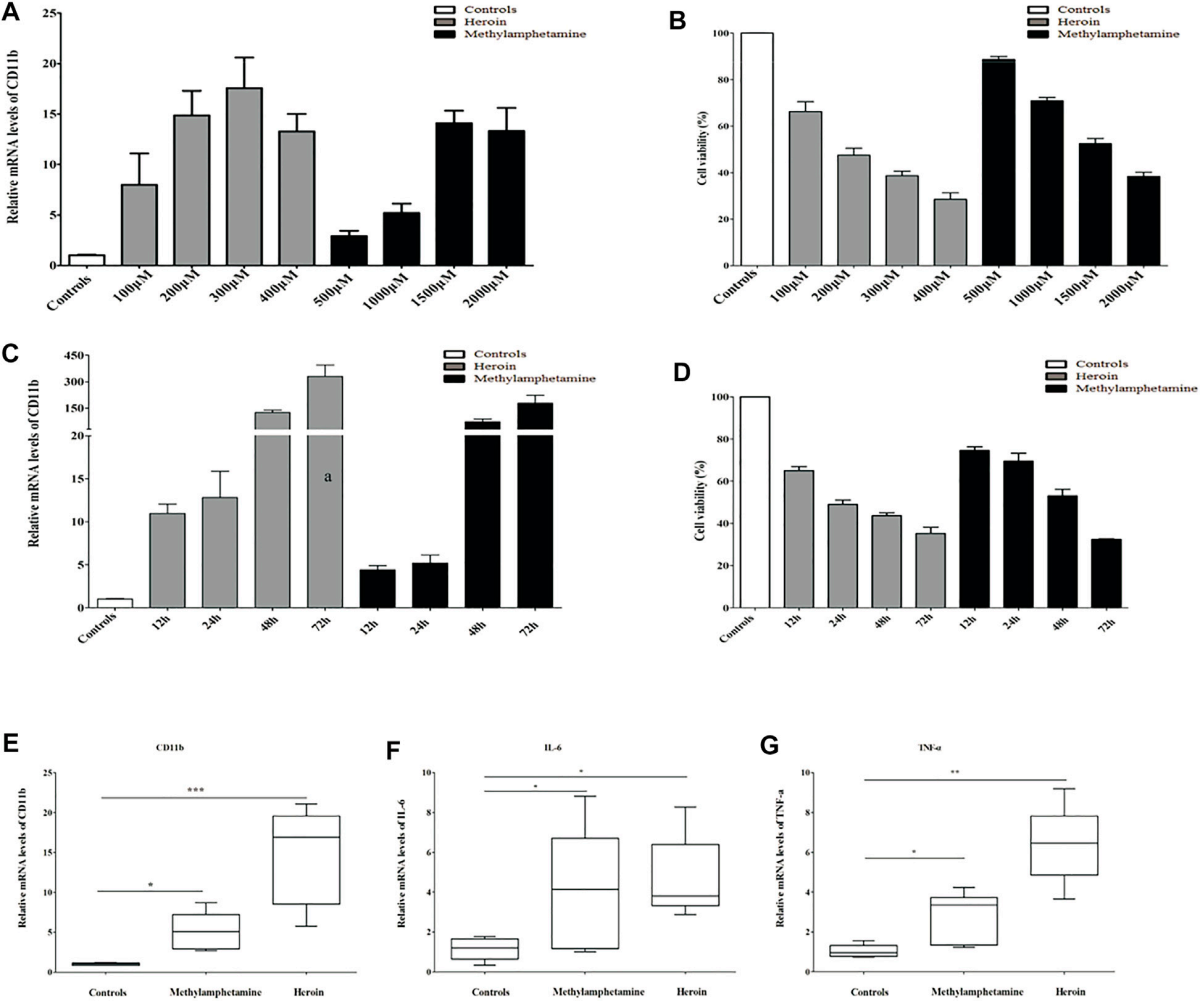
METH) and heroin increased the mRNA levels of CD11b and inflammatory cytokine in immortalized mouse microglia cells (BV2) cells. The relative mRNA levels of CD11b by qPCR and cell viability by CCK-8 assay at varied concentrations **(A, B)** and various timepoints **(C, D)** between METH and heroin, respectively. The relative mRNA levels of CD11b, IL-6, and TNF-α treated with 1,000 μM METH and 200 μM heroin after 24 h **(E–G)**, respectively. (*p < 0.05, **p < 0.01, ***p < 0.001 compared with the controls).

Immortalized mouse microglia cells (BV2) were purchased from Procell (Wuhan, China) and incubated with Dulbecco’s modified Eagle’s medium/nutrient mixture F-12 (DMEM/F12) (Gibco, USA, Catalog: 11320033) supplemented with 10% fetal bovine serum (FBS) (BI, USA, Catalog: 04-002-1A), and 1% penicillin and streptomycin (Solarbio, Beijing, China, catalog: P1400). The BV2 cells were seeded in six-well plates at a density of 2.5 × 10^5^ cells/well with 2 ml of complete medium cultured at 37°C in a humidified 5% CO_2_ atmosphere until the cell confluence reached 60%–70%. Then BV2 cells were treated with different concentrations of METH or heroin for different periods to mimic the drug injury *in vitro*, respectively. In the study, the relative mRNA levels of CD11b (a marker of activated microglia) substantially increased with METH above 500 μM and heroin above 100 μM, and with a peak response at 2,000 μM of METH and 400 μM of heroin, respectively (Figure 2A). Considering the cell activity and damage (Figure 2B), the BV2 cells were treated with 1,000 μM of METH and 200 μM of heroin after 24 h, respectively. The concentration of drugs did not induce significant cell damage, even in a time course experiment (Figures 2C, D). The concentrations were consistent with previous studies (Lai et al., 2011; Park et al., 2017; Chen X. et al., 2020). Under this condition, the relative mRNA levels of CD11b, TNF-a, and IL-6 were considerably increased (Figures 2E–G). It indicated that the METH and heroin effectively established inflammatory models *in vitro*.

The qPCR was performed to examine the difference in transcriptional levels of the hub genes and key factors of signaling pathway in BV2 cells after heroin and METH treatment. The primers and amplicon sizes of hub genes are shown in Supplementary Table S1. The qPCR was performed according to the instructions of TaKara TB Green™ Premix Ex Taq™ II by Bio-Rad CFX96TM real-time system (Foster City, CA, USA). All samples were performed in triplicate. The relative mRNA levels of hub genes were calculated by comparing the average of each target gene with the reference glyceraldehyde-3-phosphate dehydrogenase (GAPDH) in the same sample with the 2^−ΔΔCt^ method.

### 2.7 Statistical analysis

The relative mRNA levels were analyzed with SPSS for Windows software package version 13.0 (SPSS, Inc., Chicago, IL, USA). All data were presented as the mean ± standard deviation (SD) for continuous variables. The Student’s *t*-test was used if the data have a normal distribution. Otherwise, the Kruskal–Wallis test was used. A two-sided *p*-value < 0.05 was taken as the level for statistical significance. ROC analysis was performed to explore the predictive accuracy of hub genes in the GSE15774 database. AUC was used to evaluate the sensitivity and specificity of each gene. The genes with an AUC of more than 0.7 and *p*-value of less than 0.05 were used to evaluate the predictive accuracy of hub genes (Fang et al., 2021; Kang et al., 2021).

## 3 Results

### 3.1 Information of included microarrays

According to the inclusion criteria, GSE30305 (Piechota et al., 2012), GSE15774 (Piechota et al., 2010), GSE78280 (Skupio et al., 2017), GSE62346 (Mcadams et al., 2015), GSE17731 (Sanchis-Segura et al., 2009), GSE7762 (Korostynski et al., 2007), GSE12387 (Palmer et al., 2005), GSE8030 (Chin et al., 2008), and GSE19914 (Eun et al., 2010) were included in the study (Table 1). For opioid treatment, 100 opioid samples and 107 controls from the six opioid treatment databases were further analyzed, including GSE30305, GSE15774, GSE78280, GSE62346, GSE17731, and GSE7762. There were 68 ATS samples and 79 controls in five ATS-treatment databases, which included GSE30305, GSE15774, GSE12387, GSE8030, and GSE19914. Additionally, GSE30305 and GSE15774 databases simultaneously contained opioid and ATS treatments. The main administration methods were intraperitoneal (ip) injection (five studies of opioids and four studies of ATS). Single-dose treatment was used in six databases, and multidose treatment was used in three databases (dose range was 10–40 mg/kg for opioids and 2–20 mg/kg for ATS). The duration of administration was 3 to 14 days for all opioid models and three ATS models. All the tissues were involved in brain reward regions, including the striatum, hippocampal, cerebral cortex, and nucleus acumbens (NAc). As shown in Supplementary Figures S1 and S2, the midline of ATS and opioid treatments were matched by boxplot analysis, and the gene expression profiles were comfortable for further study.

**TABLE 1:** Basic information of the selected databases in the study.

GSE ID	Year	Platform	Gender (M/F) and age	Cases/controls(n)	Model parameters	Drug treatment	Tissues
GSE78280	2016	GPL6887	F, 6–10 weeks	12/12	MOR, ip, 20 mg/kg, qd, ×14 days	Subacute, single dose	Striatum
GSE62346	2015	GPL10787	M, 5–9 days	10/5	MOR, ip, 2 and 5 mg/kg, bid, ×4 days	Subacute, single dose	Hippocampal
GSE30305	2012	GPL6887	M, 8–10 weeks	24/24	METH, ip, 2–10 mg/kg, bid, ×12 days	Subacute, multiple doses	Striatum
GSE30305	2012	GPL6887	M, 8–10 weeks	24/24	Heroin, ip, 10–40 mg/kg, bid, ×12 days	Subacute, multiple doses	Striatum
GSE15774	2010	GPL6105	NA, 6–10 weeks	12/12	MOR, ip, 20 mg/kg, qod, ×7 days	Subacute, single dose	Striatum
GSE15774	2010	GPL6105	NA, 6–10 weeks	12/12	Heroin, ip, 10 mg/kg, qod, ×7 days	Subacute, single dose	Striatum
GSE15774	2010	GPL6105	NA, 6–10 weeks	12/12	METH, ip, 2 mg/kg, qod, ×7 days	Subacute, single dose	Striatum
GSE19914	2010	GPL2995	M/F, 1 days	17/17	MDMA, po, 20 mg/kg, qd, ×21 days	Chronic, single dose	Cerebral cortex
GSE17731	2009	GPL6246	M, 8 weeks	6/6	MOR, ip, 20–100 mg/kg, bid, ×7 days	Subacute, multiple doses	Striatum
GSE12387	2008	GPL81	NA, 4 weeks	12/11	METH, ip, 2 and 10 mg/kg, 1 time×1 days	Acute, single dose	NAc
GSE8030	2007	GPL339	M, 7 weeks	3/3	METH, ip, 10 mg/kg, q2h, ×7 days	Subacute, single dose	Striatum
GSE7762	2007	GPL1261	M, 8–10 weeks	24/12	MOR, ih,10–40 mg/kg, tid×5 days	Subacute, multiple doses	Striatum

Note. M/F, male/female; NA, not mentioned; MOR, morphine; METH, methylamphetamine; MDMA, 3,4-methylenedioxymethamphetamine; NAc, nucleus acumbens; ip, intraperitoneal injection; po, oral; ih, subcutaneously; qd, 1 time daily; bid, 2 times daily; qod, 2 days interval; q2h, 2 h interval; tid, 3 times daily.

### 3.2 Identification of differentially expressed genes

**FIGURE 3:**
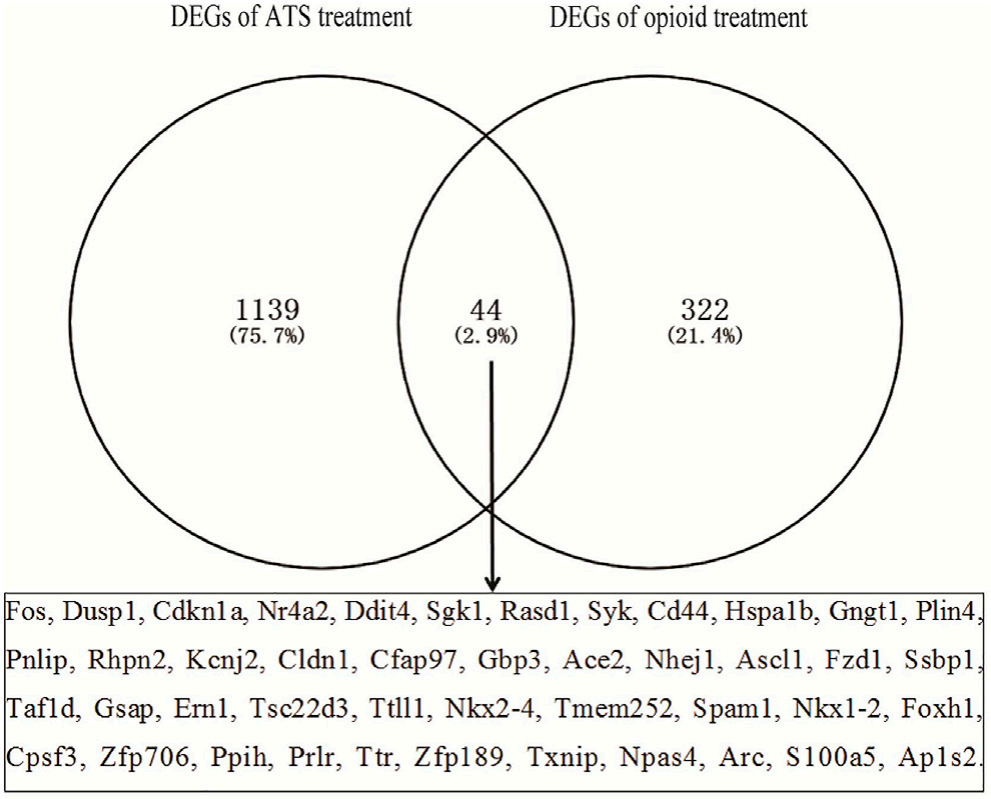
The coexpressive differentially expressed genes (CDEGs) in ATS- and opioid-treatment databases by Venn diagram.

The number of DEGs in each database by GEO2R is shown in Table 2. The number of DEGs obtained in each microarray varied widely, ranging from 0 to more than 700. There were only two DEGs for opioid treatment, but no DEGs for ATS treatment in the GSE30305 database. A total of 366 DEGs were identified from opioid treatment databases, of which 157 genes were upregulated and 209 genes were downregulated. 1,183 DEGs including 873 upregulated and 310 downregulated genes were identified from ATS treatment databases. The CDEGs between opioid and ATS treatments were identified from the all databases by Venn diagram (Figure 3). There were 44 CDEGs (17 upregulated, 8 downregulated, and 19 discordant) between opioid and ATS treatments including Fos, Ddit4, Sgk1, Dusp1, Nr4a2, Cdkn1a, Hspa1b, CD44, Gngt1, Syk, Tsc22d3, Plin4, Pnlip, Rhpn2, Cldn1, Ace2, Fzd1, Ssbp1, Ppih, Prlr, Rasd1, Txnip, Npas4, Cfap97, Ttll1, Nkx2-4, Tmem252, Foxh1, Cpsf3, Zfp706, Ap1s2, Kcnj2, Gbp3, Nhej1, Ascl1, Taf1d, Gsap, Ern1, Spam1, Nkx1-2, Ttr, Zfp189, Arc, and S100a5.

**TABLE 2:** The number of differentially expressed genes (DEGs) in each database by GEO2R.

GSE ID	Type of drugs	Total DEGs	Upregulated DEGs	Downregulated DEGs
GSE78280	Opioids	12	5	7
GSE62346	Opioids	31	8	23
GSE30305	Opioids/ATS	2/0	0/0	2/0
GSE15774	Opioids/ATS	8/17	8/17	0/0
GSE19914	ATS	367	316	51
GSE17731	Opioids	67	59	8
GSE12387	ATS	106	59	47
GSE8030	ATS	724	503	221
GSE7762	Opioids	267	92	175
Total	Opioid/ATS	366/1,183	157/873	209/310

Note. DEGs, differentially expressed genes; ATS, amphetamine-type stimulants.

### 3.3 Functional annotation, protein–protein interaction network, and interesting modules of differentially expressed genes

**FIGURE 4:**
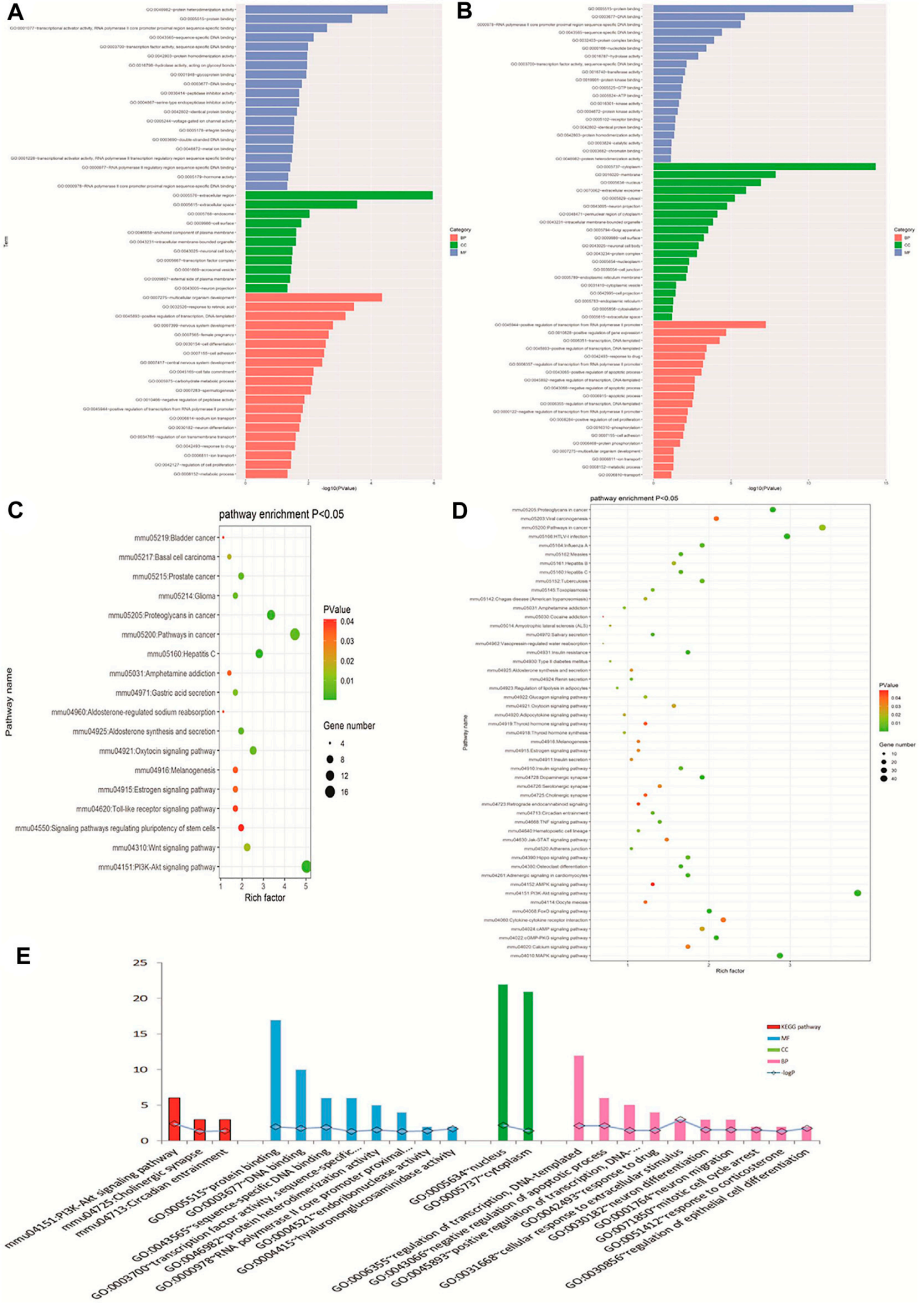
Gene Ontology (GO) and Kyoto Encyclopedia of Genes and Genomes (KEGG) pathway analyses. GO analysis of DEGs with opioid **(A)** and ATS treatments **(B)**. KEGG pathway enrichment analysis for DEGs in opioid **(C)** and ATS treatments **(D)**. GO and KEGG pathway analysis for CDEGs **(E)**.

GO biological analysis is demonstrated in Figures 4A, B. For DEGs of opioid treatment, there were about 57 terms in the biological process (BP) category, 26 terms in the molecular function (MF) category, and 11 terms in the cellular component (CC) category. Similarly, DEGs of ATS treatment were associated with 280 terms in the BP category, 96 terms in the MF category, and 92 terms in the CC category. KEGG pathway analysis indicated that the DEGs of opioid and ATS treatments were enriched in 18 and 70 signaling pathways, respectively (Figures 4C, D). Functional annotation of CDEGs indicated that 20 enriched GO terms and three KEGG pathways were detected between opioid and ATS treatments (Figure 4E). GO terms were mainly enriched in the BP category including cellular apoptotic process (GO:0043066), regulation of transcription, DNA templated (GO:0006355), neuron migration (GO:0001764) and differentiation (GO:0030182), mitotic cell cycle arrest (GO:0071850), transcriptional regulation (GO:0000122), DNA templated (GO:0045893), response to extracellular stimulus (GO:0031668), drug (GO:0042493), and corticosterone (GO:0051412) (Supplementary Table S3). KEGG pathways were mainly enriched in the PI3K/Akt signaling pathway (mmu04151), circadian entrainment (mmu04713), and cholinergic synapse (mmu04725) (Figure 4E and Supplementary Table S2).

**FIGURE 5:**
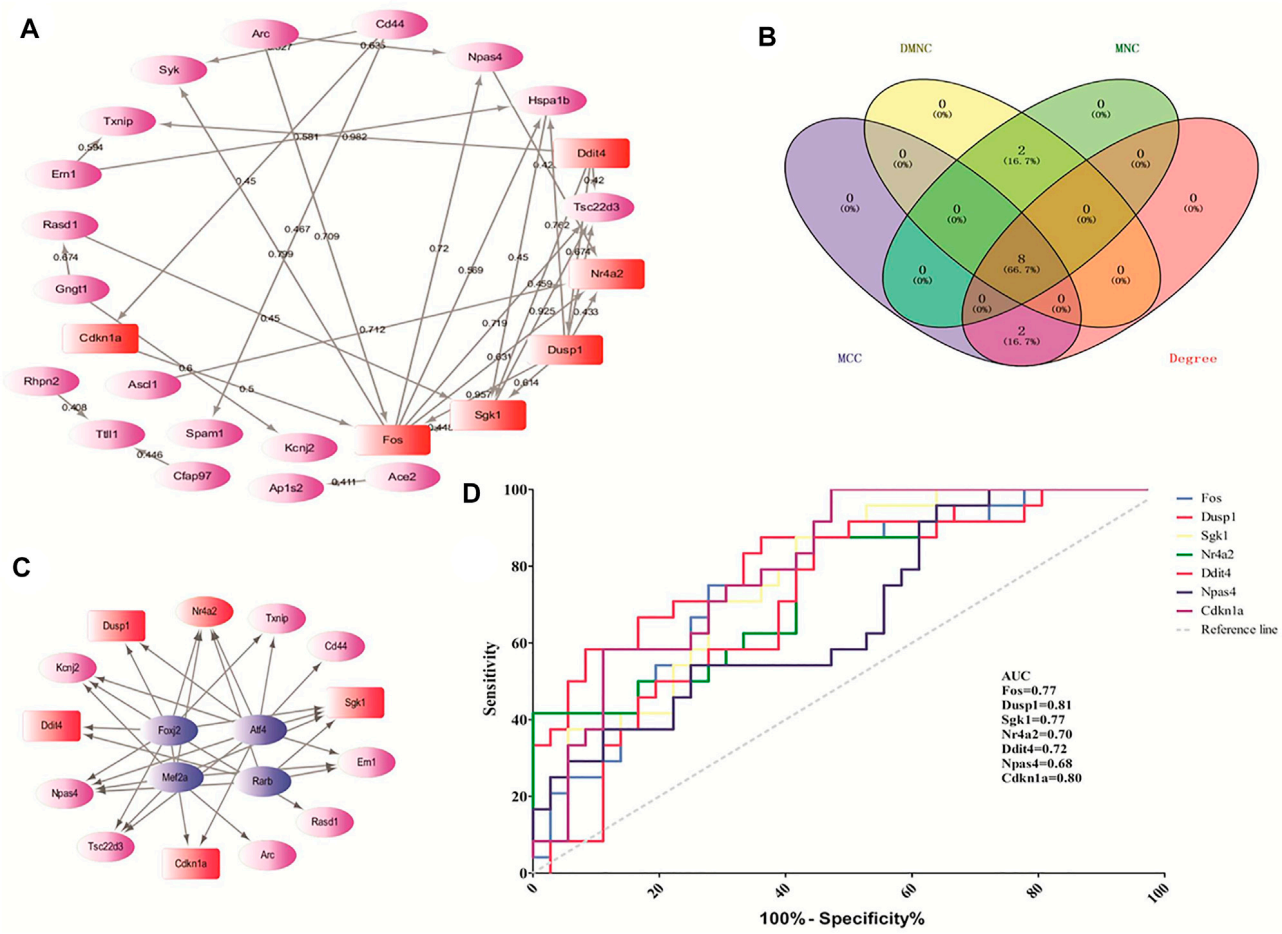
The hub genes screened by the CytoHubba plugin and predicted by receiver operating characteristic (ROC) curve. The protein–protein interaction (PPI) network of CDEGs **(A)**. Venn diagram of the top 10 hub genes by MCC, DMNC, MNC, and degree algorithms **(B)**. The PPI network of TF-target genes by the iRegulon plugin **(C)**. The ROC curve-predicted hub genes in GSE15774 **(D)**. Red indicates the hub genes. Pink indicates the other DEGs. Blue indicates TF-target genes.

According to the STRING online database, 342 nodes and 568 edges were detected in the 366 DEGs of opioid treatment, and the average node degree was 3.32. Similarly, 1,128 nodes and 5,888 edges were screened in the 1,183 DEGs of ATS treatment, and the average node degree was 10.4. A total of 44 nodes and 29 edges were screened in the 44 CDEGs of opioid and ATS treatments, and the average node degree was 1.32 (Figure 5A).

We screened 29 modules in ATS treatment, and 7 modules in opioid treatment with the MCODE plugin, respectively. In the present study, a MCODE score greater than 4.0 was considered as an interesting module (Qi et al., 2021). Finally, eight interesting modules for ATS treatment and two interesting modules for opioid treatment were selected, respectively (Table 3 and Supplementary Figure S3). Pathway enrichment analysis of the interesting module demonstrated that each module was functionally correlated (Supplementary Table S3).

**TABLE 3:** The information of interesting modules in ATS- and opioid-treatment databases, ranked in descending order of MCODE score.

Category	MCODE scores	Node/edge (n)	Gene symbol
ATS			
Module 1	18.388	67/602	Rab5b, Hspa8, Adrb2, App, Gnb2, Cxcl10, Gprc6a, Sucnr1, Drd5, Avpr2, Sh3gl1,, Gngt1, Gnaq, Crhr2, Adrb1, Gcg, Cnr1, Mgrn1, Ppbp, Wnt5a, Syt1, Lrp2, Cdc27, Sgip1, Traf7, Itsn2, Klhl25,Wasl, Dnajc6, Hc,C5ar1, Socs3, Sh3gl2, Cul7, Spsb1, Chrm3, Vamp2, Asb10, Ednra, Asb17, Il7r, Gna11, Uba7, Pik3r2, Sh3rf1, Galr1, Asb15, Cxcr5, Gnas, Tfrc, Tacr3, Gng5, Ptgdr, Ccl6, Glp1r, Btbd1, Arih2, Ccr9, Gpr45, Arpc4, Fbxw5, Tas2r119, Iapp, Anapc5, Xcl1, Gnai1,Arrb2
Module 2	10.105	57/282	Ppil1, Sf3b2, Snrpa, mTOR, Cdh1, Creb1, B2m, Trip13, Cenpa, Vwf, Ank1, Dctn1, Xab2, Lyz2, Ctsd, Snrpb2, Kif23, Snrpg, Ppih, Hnrnpa3, Pcbp1, Ctsh, Prpf4, Prg3, Serpine1, Copg1, Tk1, Copz1, Serpina1b, Mcm6, Tmed2, Gtpbp2, Prpf6, Gorasp1, Dntt, Bcas2, Maged2, Arfgap2, Cdca5, Cd55, Gosr2, Nusap1, Dynll2, Spag5, Cdc6, Ncapg, Stil, Foxo1, Actn1, Rad51ap1, Xiap, Brca1, Fen1, Cpsf3, Aldoc, Cdkn1b, Sptbn2
Module 3	7.762	43/163	Ncr1, Eef1a2, Cd40,Dusp1, Nr4a2, Cdkn1a, Hspa1b, Casp3, Mapk14, Pten, Actb, Areg, Fosb, Irf9, Il2rg, Syp, Slc1a3, Cd3e, H2-Ab1, Irgm1, Tnfsf4, Gbp2, Gbp3, Junb, Tlr7, Ctsz, Cebpb, Trim30a, Serpinb9c, Zbp1, Ntrk2, Dcx, Fosl2, Iigp1, Map2, Dlg4, Bcl2l11, Atf4, Cnih2, Egr1, Csnk1d, Cnih1, Grin2b
Module 4	7.00	7/21	Ehhadh, Gnpat, Scp2, Acot8, Crat, Acot4, Acot3
Module 5	6.415	54/170	Slamf1, Hspa5, Atf3, Fos, Tlr4, Gadd45b, Eif3c, l7Rn6, Nedd1, Ppia, Cntrl, Arc, Eif2s3x, Rps17, Eif4g2, Ppp2r5b, Ppp2r5a, Gdnf, Mmp3, Sox10, Pom121, Jak1, Prkaca, Eng, Cd38, Ptpn11, Actg1, Gata4, Cd274, Gfap, Hspa14, Il2rb, Nr4a3, Npas4, Egr2, Ppp4c, Mbp, Ppp1ca, Eif3e, Jag1, Ctla4, Ppp3r1, Lmna, Ascl1, Numa1, Rps24, Ywhag, Psmb10, Hspe1, Dnajc2, Psmd11, Rps23, Btg2, Eef2
Module 6	5.538	14/36	Cyp3a44, Apbb1, Cyp4f13, Mapk8ip1, Pla2g6, Cyp2c37, Sult2b1, Mapk8ip2, Ugt3a1, Dab1, Kif5c, Pla2g2d, Cyp2c39, Cyp2c68
Module 7	4.571	14/29	Eif2d, Wdr46, Ddx49, Eif4g1, Eif4a1, Nol12, Rps6kb2, Gnl3l, Dhx32, Eif4ebp2, Rrp7a, Trmt2a, Zfp593, Eif5a
Module 8	4.300	20/41	Ccnd3, Ccnb1, Klf2, Cd44, Stat3, Cd24a, Tbpl1, Irs2, Tcea3, Rab17, Gdi1, Rab2a, Gtf2a1, Slc2a4, Rab6b, Cd34, Ercc2, Klf4, Vim, Gtf2h4
Opioids			
Module 1	7.167	13/43	Spp1, Cd44, Trp53, Egfr, Igf2, Kdr, Sox9, Serpinc1, Ins1, Prss23, F5, Ktn1, Dmp1
Module 2	4.947	19/43	Zic1, Pax6, Slc17a7, Fos, Afp, Fzd1, Lpar3, Fzd2, Vangl1, Wnt9a, Nts, Wnt3, Oxt, P2ry13, Ptgdr2, Calb2, Ascl1, Gngt1, Olig2

Note. ATS, amphetamine-type stimulants.

### 3.4 Hub genes screened by CytoHubba plugin and molecular complex detection plugin

According to the CytoHubba plugin with MCC algorithm in Cytoscape, the hub genes were significantly different between opioid- and ATS-treatment DEGs (Supplementary Table S4). The Fos, Dusp1, Sgk1, Nr4a2, Ddit4, Hspa1b, Npas4, Cdkn1a, Cd44, and Rasd1 were identified as hub genes (Table 4). Similar results were obtained by DMNC, MNC, and degree algorithms (Table 4 and Figure 5B).

**TABLE 4:** Maximal clique centrality (MCC), density of maximum neighborhood component (DMNC), maximum neighborhood component (MNC), and degree score of the top 10 hub genes by the CytoHubba plugin in Cytoscape Software.

Rank	MCC		DMNC		MCN		Degree	
	Gene symbol	Score	Gene symbol	Score	Gene symbol	Score	Gene symbol	Score
1	Fos	13	Npas4	0.309	Fos	7	Fos	8
2	Dusp1	10	Nr4a2	0.309	Dusp1	6	Dusp1	6
3	Sgk1	8	Hspa1b	0.308	Sgk1	5	Sgk1	5
4	Nr4a2	5	Arc	0.308	Npas4	3	Hspa1b	4
5	Ddit4	4	Tsc22d3	0.308	Nr4a2	3	Nr4a2	4
6	Hspa1b	4	Cdkn1a	0.308	Hspa1b	2	Ddit4	4
7	Npas4	4	Ddit4	0.308	Arc	2	Npas4	3
8	Cdkn1a	3	Sgk1	0.259	Tsc22d3	2	Cd44	3
9	Cd44	3	Dusp1	0.238	Cdkn1a	2	Cdkn1a	3
10	Rasd1	2	Fos	0.220	Ddit4	2	Rasd1	2

Based on the MCODE score, we selected the top three targets in each interesting module as the hub genes. In opioid treatment, SPP1, CD44, Trp53, (module 1), Zic1, Pax6, and Slc17a7 (module 2) were screened as hub genes. Similarly, Rab5b, Hspa8, Adrb2 (module 1), Ppil1, Sf3b2, Snrpa (module 2), Ncr1, Eef1a2, CD40 (module 3), Ehhadh, Gnpat, Scp2 (modul4), Slafm1, Hspa5, Atf3 (module 5), Cyp3a44, Apbb1, Cyp4f13 (module 6), Eif2d, Wdr46, Ddx49 (module 7), Ccnd3, Ccnb1, and Klf2 (module 8) were hub genes for ATS treatment (Supplementary Figure S3). Furthermore, most hub genes screened by the CytoHubba plugin were also enriched in the interesting modules.

We found four TFs (Foxj2, Alf4, Rarb, and Mef2a) with NES ＞ 4.5, which may regulate the expression of the top 20 hub genes. These TF-target genes could regulate the expression of Dusp1, Sgk1, Ddit4, Nr4a2, and Cdkn1a (Figure 5C).

### 3.5 Receiver operating characteristic curves predicted accuracy of hub genes in the GSE15774 database

In order to verify the predictive accuracy of hub genes, ROC curve analysis was performed in GSE15774. The relative mRNA levels of hub genes in GSE15774 are shown in Table 5. Among the hub genes of ATS- and opioid-treatment databases, the Fos, Dusp1, Sgk1, Ddit4, Npas4, and Cdkn1a mRNA levels were increased, while the Nr4a2 mRNA levels were decreased. Rasd1 mRNA levels in opioid treatment were different from controls, but not in ATS treatment. There were no significant difference in CD44 and Hspa1b mRNA levels in neither ATS nor opioid treatment than those of controls (Table 5). ROC analysis indicated that AUC of Fos, Dusp1, Sgk1, Nr4a2, Ddit4, and Cdkn1a was more than 0.7 and *p *＜ *0*.*05* in the GSE15774 database (Figure 5D). It might be potential hub genes for ATS and opioid dependence and needed to be verified by qPCR in BV2 cells.

**TABLE 5:** The relative mRNA levels of hub genes in GSE15774 database (mean ± SD).

Gene symbol	METH	Opioids	Controls	*p* ^1^	*p* ^2^
Fos	8.46 ± 0.51	8.32 ± 0.40	7.97 ± 0.35	0.005*	0.002
Dusp1	11.44 ± 0.52	11.26 ± 0.28	10.87 ± 0.34	0.001*	＜0.001
Sgk1	9.69 ± 0.51	9.84 ± 0.53	9.28 ± 0.34	0.007	0.001*
Nr4a2	8.36 ± 0.35	8.49 ± 0.34	8.69 ± 0.30	0.012	0.003
Ddit4	8.98 ± 0.47	9.04 ± 0.55	8.66 ± 0.25	0.035*	0.008*
Hspa1b	6.81 ± 0.23	6.82 ± 0.18	6.78 ± 0.20	0.729	0.524
Npas4	7.42 ± 0.35	7.31 ± 0.25	7.16 ± 0.20	0.041*	0.026
Cdkn1a	9.40 ± 0.41	9.46 ± 0.49	8.95 ± 0.27	0.002*	＜0.001
CD44	6.49 ± 0.05	6.49 ± 0.05	6.48 ± 0.03	0.330	0.455
Rasd1	7.54 ± 0.40	7.64 ± 0.37	7.29 ± 0.27	0.087*	0.001

Note. *p* ^1^, METH vs. controls; *p* ^2^, opioids vs. controls; *Kruskal–Wallis test.

### 3.6 The quantitative real-time PCR verified the mRNA levels of hub genes

**FIGURE 6:**
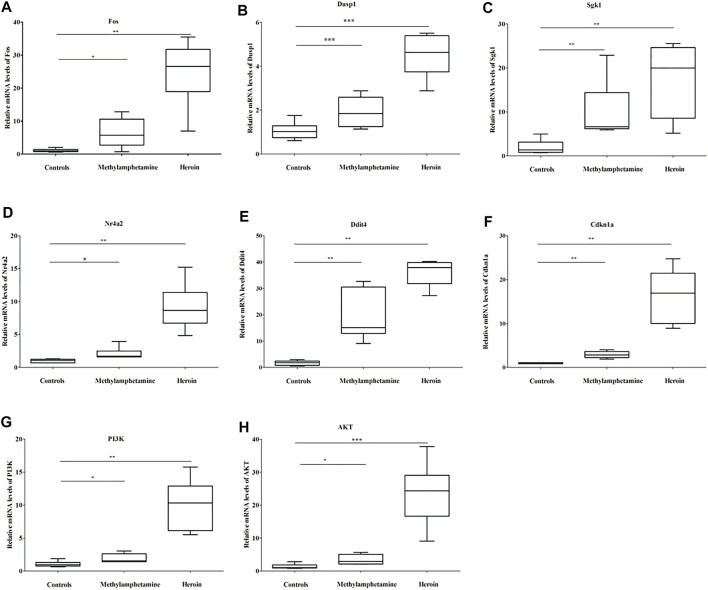
The relative mRNA levels of Fos **(A)**, Dusp1 **(B)**, Sgk1 **(C)**, Nr4a2 **(D)**, Ddit4 **(E)**, Cdkn1a **(F)**, PI3K **(G)**, and Akt **(H)** in BV2 cells treated with 1,000 μM METH and 200 μM heroin after 24 h, respectively (^*^ *p* ＜ 0.05, ^**^ *p* ＜ 0.01, ^***^ *p* ＜ 0.001 compared with the controls).

The relative mRNA levels of Fos, Dusp1, Sgk1, Ddit4, Cdkn1a, PI3K, and Akt in BV2 cells in both METH and heroin treatments significantly increased compared with those of controls (*p *＜ *0*.*05*), respectively (Figure 6 and Supplementary Table S5). The results were consistent with those of the bioinformatic analysis and GSE15774 database. However, the Nr4a2 mRNA level increased in BV2 cells, while it decreased in the bioinformatic analysis and GSE15774 database.

## 4 Discussion

The bioinformatic analysis was conducted to better understand the hub genes and molecular mechanisms of substance dependence. In the study, 44 CDEGs were identified between ATS and opioid databases. The top 10 hub genes were mainly enriched in apoptotic process (CD44, Dusp1, Sgk1, and Hspa1b), neuron differentiation, migration, and proliferation (Nr4a2 and Ddit4), response to external stimulation (Fos and Cdkn1a), and transcriptional regulation (Nr4a2 and Npas4). They were also prominently enriched in the PI3K/Akt signaling pathway. The relative mRNA levels of the aforementioned hub genes were significantly different between cases and controls in the GES15774 database. ROC analysis found that the AUC scores of hub genes (Fos, Dusp1, Sgk1, Nr4a2, Ddit4, and Cdkn1a) were more than 0.70 in GSE15774. It indicated that the hub genes could be accurately predicted. The mRNA levels of Fos, Dusp1, Sgk1, Ddit4, Cdkn1a, PI3K, and AKT were significantly increased in BV2 cells with METH and heroin treatments, respectively. However, the Nr4a2 mRNA levels increased in BV2 cells, and decreased in bioinformatic analysis. These results were consistent with those of the bioinformatic analysis and GSE15774 database.

In the bioinformatic analysis, nine databases (six studies of opioid treatment, and four studies of ATS treatment) were downloaded from the GEO database, and GSE30305 and GSE15774 databases simultaneously contained opioid and ATS treatments. The number of DEGs obtained in each microarray ranged from 0 to more than 700. It might be caused by the differences in detection platform and model parameters including drug types, doses, frequency, and duration of drug administration. GEO2R analysis indicated that a total of 366 DEGs including 157 upregulated and 209 downregulated DEGs in opioid treatment, and 1,183 DEGs including 873 upregulated and 310 downregulated DEGs in ATS treatment were identified, respectively. BP was the main enriched term of those DEGs, including transcriptional regulation, apoptotic process, phosphorylation, cell proliferation and adhesion, and nervous system development in ATS treatment, and nervous system development, transcriptional regulation, cell differentiation and adhesion, and ion transport in opioid treatment. It indicated that the common biological processes involved in ATS and opioid dependencies were apoptosis, nervous system development, cell differentiation, and proliferation (Piechota et al., 2010; Piechota et al., 2012). In the KEGG pathways, the PI3K/Akt signaling pathway was the prominently enriched pathway in either opioid or ATS treatment. Furthermore, there were 44 CDEGs between ATS- and opioid-treatment databases. The results illustrated that ATS and opioid dependencies shared common target genes and molecular mechanism, which were consistent with the previous studies and whole-genome microarray profiling (Piechota et al., 2012; Korostynski et al., 2013; Lopez-Leon et al., 2021).

The PI3K/Akt signaling pathway was the main enriched signaling pathway between ATS and opioid treatments, and was involved in regulating the process of cell survival, growth, proliferation, angiogenesis, transcription, translation, metabolism, and apoptosis (Gaesser and Fyffe-Maricich 2016; Sinha et al., 2018; Ediriweera et al., 2019). Some studies indicated that the PI3K/Akt signaling pathway played an essential role in neuronal survival (Dudek et al., 1997) and neurodegeneration (Wu et al., 2010; Chen et al., 2012). Qiao et al. reported that drugs (e.g., alcohol, heroin, morphine, and METH) could activate the PI3K/Akt signaling pathway in the cortex, and contribute to addiction (Qiao et al., 2018; Li et al., 2020; Meng et al., 2020; Zhu et al., 2021). The Akt phosphorylation in the NAc was related to heroin-seeking behavior (Zhu et al., 2021). Furthermore, we detected that the PI3K and Akt mRNA levels in BV2 cells treated with METH and heroin significantly increased than those of controls. It showed that the PI3K/Akt signaling pathway was the common signaling pathway and played vital roles in ATS and opioid treatments. Interestingly, Sgk1, Ddit4, and Cdkn1a (hub genes) were also enriched in the PI3K/Akt signaling pathway. Sgk1, Ddit4, and Cdkn1a were located in 6q23.2, 10q24.33, and 6p21.2 and encoded at 49 kD with 431 amino acids, 25 kD with 232 amino acids, 18.1 kD with 164 amino acids, respectively (Demetrick et al., 1995; Kobayashi et al., 1999; Ellisen et al., 2002). The hub genes were widely distributed in different tissues, and were involved in multiple physiological functions and pathophysiological conditions, such as hypoxia, ionizing radiation, heat shock, oxidative stress, hormone release, cell proliferation and apoptosis, autophagy, fibrosis disease, ischemia sequelae, neuronal survival, neuroexcitability, and neurodegeneration (Ellisen et al., 2002; Shoshani et al., 2002; Malagelada et al., 2006; Anacker et al., 2013; Canal et al., 2014; Wolff et al., 2014; Jin et al., 2019; Fattahi et al., 2021). The Sgk1, Ddit4, and Cdkn1a mRNA levels in the databases significantly increased, except Sgk1 in GSE78280. The GSE78280 database was the only female model, the difference might be associated with gender (Dumeige et al., 2017). Further study is needed to reveal the exact reason. The relative mRNA levels of Sgk1, Ddit4, and Cdkn1a markedly increased in BV2 cells with METH and heroin treatments than those of controls. The results were consistent with the bioinformatic analysis and other studies on liver injury, traumatic brain injury, anterior cruciate ligament transection, and lung injury (Lang et al., 2008; Chen et al., 2016; Steiner et al., 2016; Wong et al., 2016; Li et al., 2017; Faust et al., 2020; Blazquez-Prieto et al., 2021; Guillot et al., 2021). The data demonstrated that Sgk1, Ddit4, and Cdkn1a might play vital roles in ATS and opioid dependencies (Piechota et al., 2010). Furthermore, function annotation demonstrated that Sgk1, Ddit4, and Cdkn1a were mainly involved in apoptotic process and cell proliferation. It suggested that Sgk1, Ddit4, and Cdkn1a played key roles in ATS and opioid dependencies through neuronal autophagy and apoptosis, and may be potential target genes of drug dependence. Combined with the aforementioned results, we speculated that the PI3K/Akt signaling pathway and related genes could regulate the pathogenesis of drug dependence though autophagy and apoptosis.

With Fos and Dusp1, as the immediate early genes (Wojcieszak et al., 2019), the relative mRNA levels increased in opioid- and most ATS-treatment databases. The results were in line with other studies [e.g., heroin, cocaine, △9-tetrahydrocannabinol (THC), and amphetamine] in different brain reward regions (e.g., NAc, neocortex, posterior caudate, and striatum) (Singh et al., 2005; Mattson et al., 2007; Paolone et al., 2007; Celentano et al., 2009; Fanous et al., 2012; Rubio et al., 2015; Wojcieszak et al., 2019). Fos and Dusp1 locus resided on 14q21–q31 and 5q35.1, encoded approximately 380 and 367 amino acids, respectively (Visvader et al., 1988). Fos and Dusp1 played vital roles in regulating signaling transduction, cell proliferation, and differentiation (Duric et al., 2010). Some studies demonstrated that Fos and Dusp1 were not only deemed as biomarkers of neuronal activity (Hughes and Dragunow 1995) but also a vital initial step in regulating neuroplasticity caused by drugs (Harlan and Garcia 1998). Kuroda et al. even demonstrated that Fos played a neuroprotective role in the progress of neurotoxicity caused by methamphetamine (Kuroda et al., 2010). In the present study, the relative mRNA levels of Fos and Dusp1 also increased in BV2 cells treated with METH and heroin, which was consistent with the study of Takaki et al. in acute METH administration (Takaki et al., 2001; Wojcieszak et al., 2019). However, Beauvais et al. reported that repeated injection of METH did not alter the Fos mRNA levels (Beauvais et al., 2010). It may be that Fos and Dusp1 were the immediate early genes. Interestingly, the mRNA levels of Fos and Dusp1 decreased in the GSE19914. It might be associated with drug species. GSE19914 mainly focused on MDMA and newborns (one pup), while other databases were prominent METH and young/adult mice.

Nr4a2 was predominantly expressed in the midbrain, substantia nigra, and ventral tegmental (Zetterstrom et al., 1997; Torii et al., 1999), which played an essential role in protecting dopaminergic neurons and limiting proinflammatory neurotoxin. Nr4a2 (−/−) mice failed to synthesize brain dopaminergic neurons, which resulted in mice hypoactivity, and died rapidly after birth (Saijo et al., 2009). In the study, Nr4a2 was mainly involved in neuron differentiation and migration, response to external stimulation, and transcriptional regulation (Horvath et al., 2007). Combined with previous studies, we speculated that the Nr4a2 was a potential target gene for substance dependence. However, the Nr4a2 mRNA levels were increased in BV2 cells with METH and heroin treatments, while they decreased in the bioinformatic analysis and GSE15774 database. The qPCR was performed only on BV2 cells, and microarrays were from the C57BL/6 brain musculus, which contained neurons, astrocytes, microglia, and neural intermediants.

There were some limitations in the present study. First, due to database limitations, the microarrays with different model parameters were all included. It might result in biases of selected hub genes. So, the hub genes might not really be involved in the pathogenesis of substance dependence. Further studies are needed to verify the pathophysiological mechanism of these hub genes and pathways that participated in the substance-dependent animal model and humans. Second, the study was based on animal databases and BV2 cells. The results might not be suitable to extrapolate to substance dependence of other animal models and humans. Despite the interspecies differences, animal studies contributed significantly to addiction research and are still of great assistance for future research with a more relevant model of compulsive drug use in humans. Third, the mechanisms of ATS and opioid dependencies might involve both genetic and epigenetic aspects. Epigenetic regulation consequences were beyond alterations in steady-state levels of expressed RNAs, which were far beyond the coverage of microarrays. The next-generation sequencing technologies (e.g., ChIP-seq and RNA-seq) could provide additional information. That work would be the focus of our future studies.

In conclusion, Fos, Dusp1, Sgk1, Nr4a2, Ddit4, and Cdkn1a hub genes were associated with ATS and opioid dependencies. Functional annotation and KEGG pathway analysis suggested that apoptosis, neuron differentiation, migration and proliferation, and the PI3K/Akt signaling pathway might play a critical role in pathogenesis of drug dependence. The findings may be helpful for better understanding of the shared pathogenesis and molecular mechanisms of ATS and opioid dependencies, and consequently detecting the new detection and potential therapeutic targets for drug dependence.

## Data Availability

The original contributions presented in the study are included in the article/Supplementary Material, further inquiries can be directed to the corresponding authors.
