# Supplementary material for: Identification and Verification of Potential Hub Genes in Amphetamine-Type Stimulant (ATS) and Opioid Dependence by Bioinformatic Analysis
Source: Front Genet. 2022 Mar 30;13:837123. doi: 10.3389/fgene.2022.837123 (PMC9006114; doi:10.3389/fgene.2022.837123)
Supplement: Supplementary file 1 [file DataSheet1.doc]

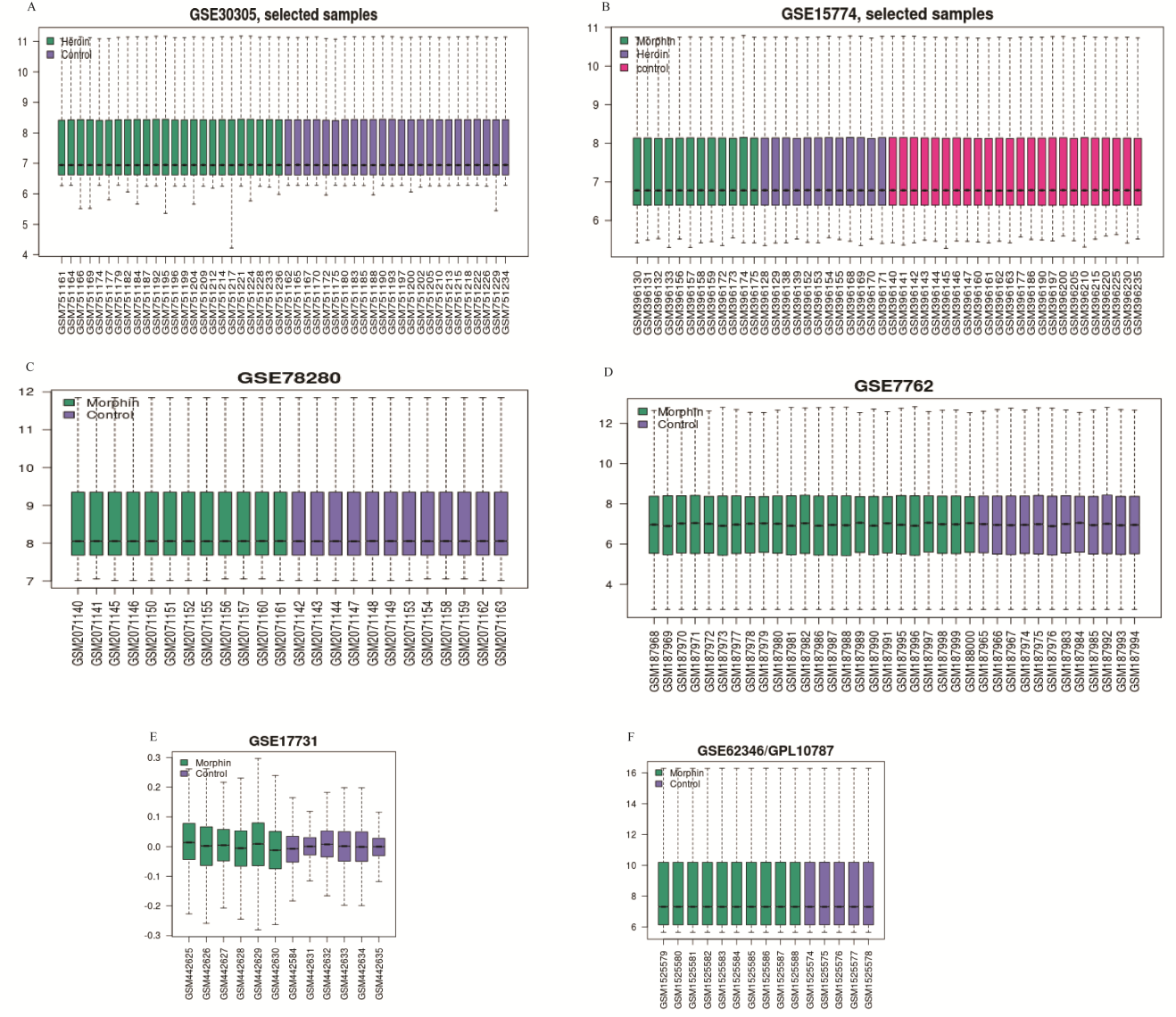


Fig S1 Distribution of gene expression profiles in opioid treatment and controls from GEO databases


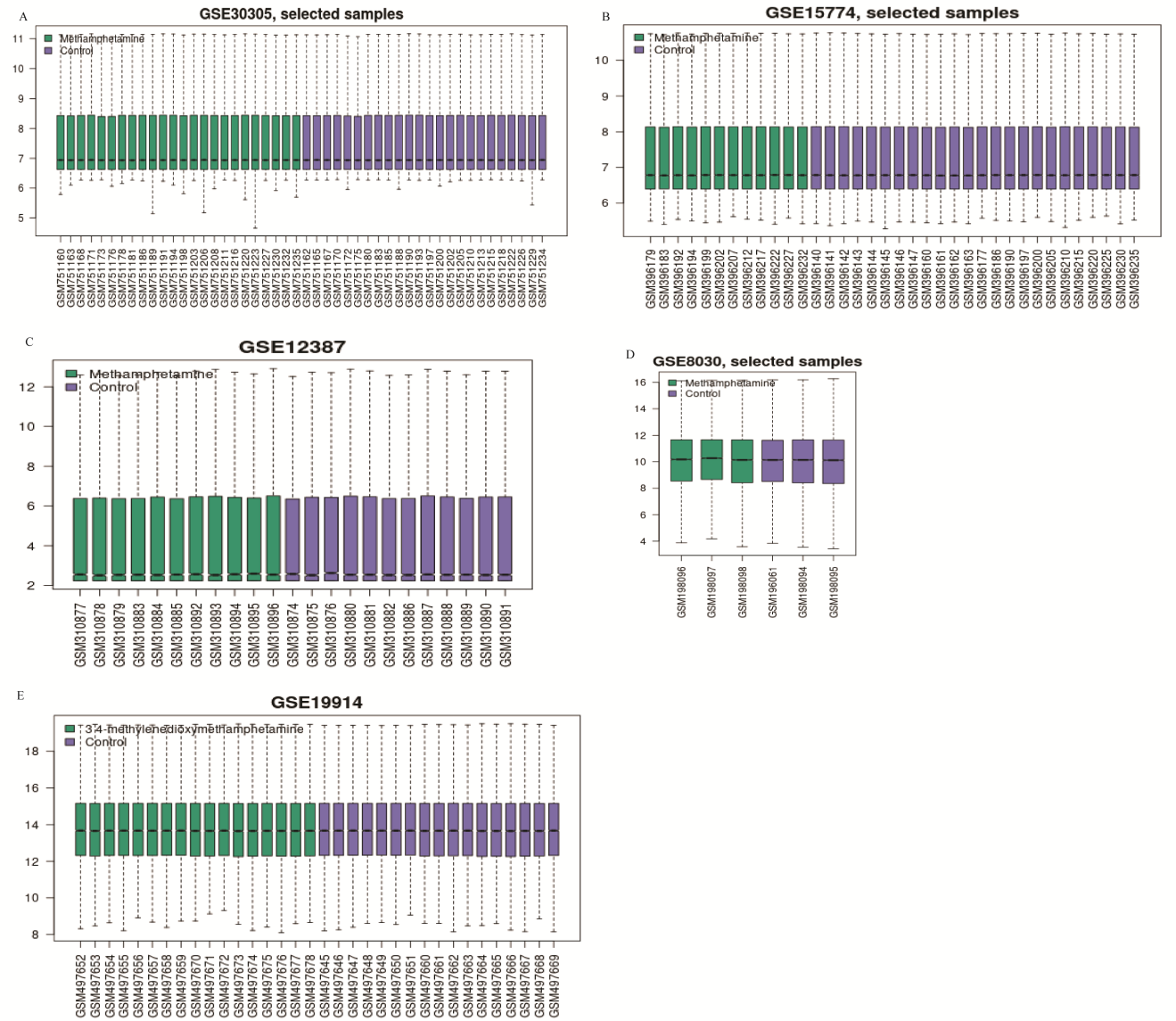


Fig S2 Distribution of gene expression profiles in ATS treatment and controls from GEO databases


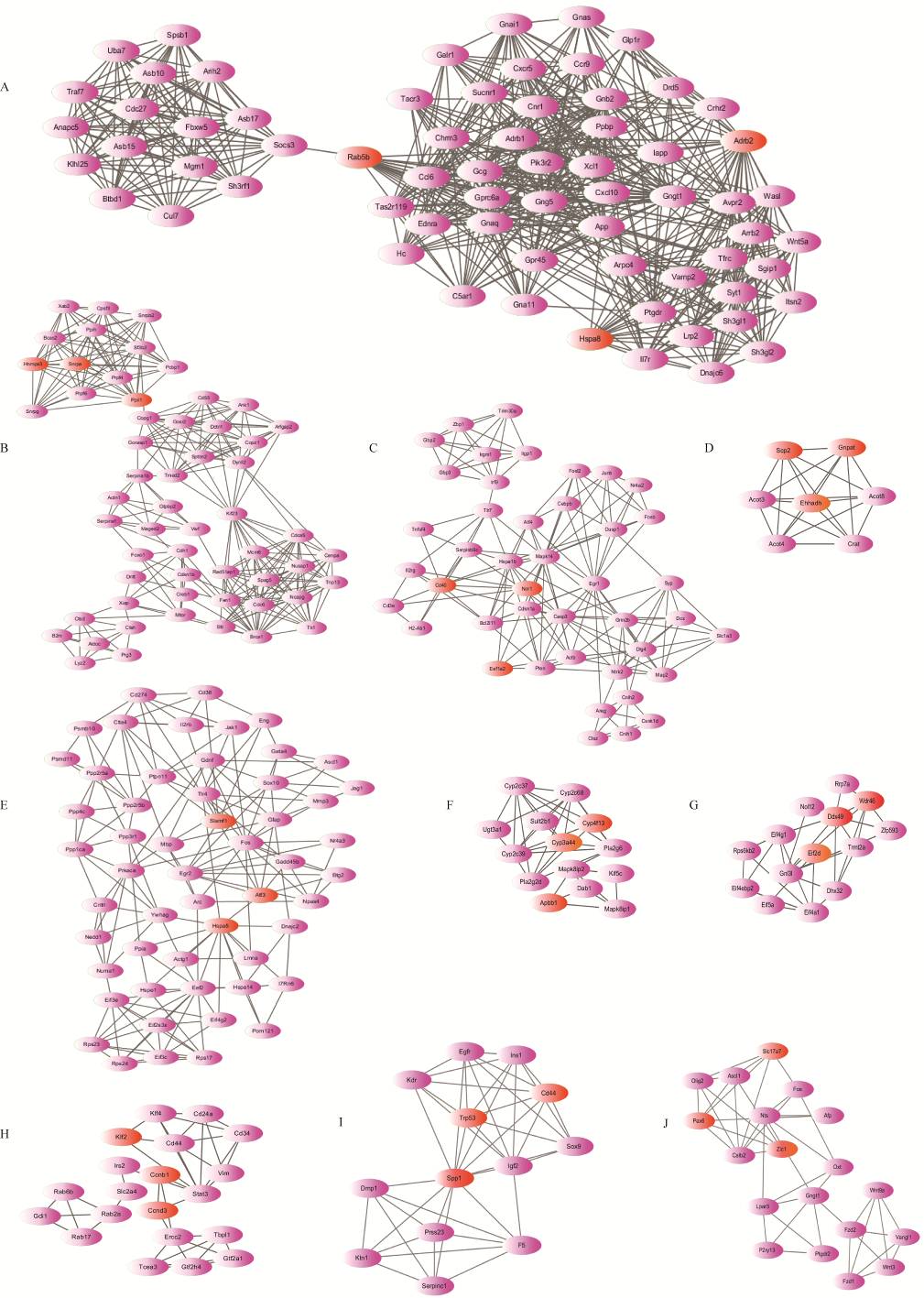


Fig S3 The PPI Network of interesting modules

Interesting module of ATS treatment (A-H). Interesting module of opioid treatment (I-J).The red indicated the hub gene. The pink indicated the other DEGs.

Table S1 Primers of hug genes of quantitative real-time polymerase chain reaction (qPCR)

| Gene symbol | Forward (5' to 3') | Reverse (5' to 3') | Size (bp) |
| --- | --- | --- | --- |
| *Fos* | TGAAGATGAGAAGTCTGCGTTGC | CCTCTGGGAAGCCAAGGTCAT | 128 |
| *Dusp1* | TTGTGAAGCAGAGGCGGAGTA | GATGGAAACAGGGAAGTTGAAGA | 176 |
| *Sgk1* | TGGGCTATCTGCACTCCCTAAAC | AGAAGGTAGATGTTGTCCCGTTATG | 144 |
| *Nr4a2* | TAGCCCGATGTGGGACGAT | AGAAGAGTGACAGGCGGGAGAC | 113 |
| *Ddit4* | AGTTGACCCTGGTGCTGCGT | GCTGCTCGGAGCTGTAGAGTTTC | 159 |
| *Cdkn1a* | GTCCAATCCTGGTGATGTCCG | AGTCAAAGTTCCACCGTTCTCG | 152 |
| *PI3K* | TGAAGAACAATGCCAAACCCA | ACTGTCAATGATCTCACTGATACGC | 142 |
| *AKT* | ATCGTCGCCAAGGATGAGG | GGTCGTGGGTCTGGAATGAGT | 109 |
| *CD11b* | CTTCCAGGGCAGGAGTCGTA | TGAGAGCCAAGAGCACCAGG | 124 |
| *IL-6* | CCTTCTTGGGACTGATGCTGG | CCACGATTTCCCAGAGAACATG | 153 |
| *TNF-a* | AGCCGATGGGTTGTACCTTGT | ACGGCAGAGAGGAGGTTGACT | 135 |
| *GAPDH* | GACATCAAGAAGGTGGTGAAGC | GAAGGTGGAAGAGTGGGAGTT | 117 |

Table S2 The Gene Ontology (GO) and Kyoto Encyclopedia of Genes and Genomes (KEGG) pathway analysis of CDEGs

| Term | Richfactor | P | Gene symbol |
| --- | --- | --- | --- |
| Pathway |  |  |  |
| mmu04151:PI3K-Akt signaling pathway | 13.64 | 0.004 | *GNGT1, CDKN1A, SYK, DDIT4, SGK1, PRLR* |
| mmu04713:Circadian entrainment | 6.82 | 0.037 | *GNGT1, RASD1, FOS* |
| mmu04725:Cholinergic synapse | 6.82 | 0.048 | *GNGT1, FOS, KCNJ2* |
| MF |  |  |  |
| GO:0005515~protein binding | 38.64 | 0.011 | *CDKN1A, SYK, FOXH1, ASCL1, CLDN1, PRLR, NR4A2, ERN1, TTR, TTLL1, NKX1-2, AP1S2, TXNIP, SGK1, KCNJ2, HSPA1B, CD44* |
| GO:0043565~sequence-specific DNA binding | 13.64 | 0.013 | *NR4A2, FOXH1, NKX1-2, FOS, ASCL1, NKX2-4* |
| GO:0003677~DNA binding | 22.73 | 0.018 | *NPAS4, NR4A2, TAF1D, FOXH1, NKX1-2, SSBP1, FOS, ASCL1, NKX2-4, NHEJ1* |
| GO:0004415~hyalurononglucosaminidase activity | 4.55 | 0.020 | *SPAM1, CD44* |
| GO:0046982~protein heterodimerization activity | 11.36 | 0.027 | *NPAS4, NR4A2, FZD1, TTR, FOS* |
| GO:0004521~endoribonuclease activity | 4.55 | 0.040 | *ERN1, CPSF3* |
| GO:0003700~transcription factor activity, sequence-specific DNA binding | 13.64 | 0.046 | *NR4A2, TSC22D3, FOXH1, FOS, ASCL1, ZFP189* |
| GO:0000978~RNA polymerase II core promoter proximal region sequence-specific DNA binding | 9.09 | 0.046 | *NPAS4, NR4A2, FOS, ASCL1* |
| CC |  |  |  |
| GO:0005634~nucleus | 50.00 | 0.007 | *CDKN1A, SYK, DUSP1, TSC22D3, CPSF3, FOXH1, ZFP706, FOS, ASCL1, NPAS4, NR4A2, TAF1D, RASD1, NKX1-2, S100A5, TXNIP, SSBP1, SGK1, NKX2-4, NHEJ1, CD44, GBP3* |
| GO:0005737~cytoplasm | 47.73 | 0.043 | *CDKN1A, SYK, DUSP1, TSC22D3, ZFP706, FOS, CLDN1, NR4A2, ERN1, ACE2, ARC, RASD1, TTLL1, DDIT4, PLIN4, TXNIP, PPIH, RHPN2, SGK1, CD44, GBP3* |
| BP |  |  |  |
| GO:0031668~cellular response to extracellular stimulus | 6.82 | 0.001 | *NR4A2, CDKN1A, FOS* |
| GO:0043066~negative regulation of apoptotic process | 13.64 | 0.007 | *CDKN1A, DUSP1, ASCL1, SGK1, HSPA1B, CD44* |
| GO:0006355~regulation of transcription, DNA-templated | 27.27 | 0.007 | *NPAS4, NR4A2, ERN1, TAF1D, TSC22D3, FOXH1, NKX1-2, TXNIP, FOS, ASCL1, ZFP189, NKX2-4* |
| GO:0030856~regulation of epithelial cell differentiation | 4.55 | 0.017 | *ASCL1, PRLR* |
| GO:0001764~neuron migration | 6.82 | 0.029 | *NR4A2, DDIT4, ASCL1* |
| GO:0071850~mitotic cell cycle arrest | 4.55 | 0.030 | *CDKN1A, DUSP1* |
| GO:0030182~neuron differentiation | 6.82 | 0.031 | *NR4A2, DDIT4, ASCL1* |
| GO:0045893~positive regulation of transcription, DNA-templated | 11.36 | 0.035 | *NPAS4, NR4A2, FZD1, FOXH1, FOS* |
| GO:0042493~response to drug | 9.09 | 0.036 | *FZD1, CDKN1A, TXNIP, FOS* |
| GO:0051412~response to corticosterone | 4.55 | 0.044 | *CDKN1A, FOS* |

BP, biological process; MF, molecular function; CC,cellular component.

Table S3 Kyoto Encyclopedia of Genes and Genomes (KEGG) pathway enrichment analysis of interesting modules.

| Term | Richfactor | -LogP | Gene Symbol |
| --- | --- | --- | --- |
| ATS treatment |  |  |  |
| Module 1 |  |  |  |
| mmu04062:Chemokine signaling pathway | 19.40 | 8.34 | *CXCR5, PIK3R2, PPBP, ARRB2, WASL, GNAI1, GNGT1, CXCL10, GNG5, CCL6, GNB2, CCR9, XCL1* |
| mmu04080:Neuroactive ligand-receptor interaction | 19.40 | 6.54 | *GLP1R, CHRM3, AVPR2, C5AR1, TACR3, ADRB1, ADRB2, CRHR2, EDNRA, CNR1, GALR1, PTGDR, DRD5* |
| mmu05200:Pathways in cancer | 14.93 | 2.93 | *GNGT1, EDNRA, GNG5, GNA11, GNB2, GNAQ, WNT5A, GNAS, PIK3R2, GNAI1* |
| mmu04020:Calcium signaling pathway | 13.43 | 4.62 | *CHRM3, EDNRA, GNA11, GNAQ, GNAS, TACR3, ADRB1, ADRB2, DRD5* |
| mmu04024:cAMP signaling pathway | 13.43 | 4.34 | *GLP1R, EDNRA, SUCNR1, GNAS, ADRB1, PIK3R2, ADRB2, GNAI1, DRD5* |
| mmu04144:Endocytosis | 13.43 | 3.49 | *HSPA8, RAB5B, TFRC, DNAJC6, ARPC4, ARRB2, WASL, SH3GL2, SH3GL1* |
| mmu04725:Cholinergic synapse | 11.94 | 5.02 | *GNGT1, CHRM3, GNG5, GNA11, GNB2, GNAQ, PIK3R2, GNAI1* |
| mmu04911:Insulin secretion | 10.45 | 4.66 | *GLP1R, CHRM3, GNA11, GNAQ, GNAS, GCG, VAMP2* |
| mmu04726:Serotonergic synapse | 10.45 | 3.61 | *GNGT1, APP, GNG5, GNB2, GNAQ, GNAS, GNAI1* |
| mmu04728:Dopaminergic synapse | 10.45 | 3.58 | *GNGT1, GNG5, GNB2, GNAQ, GNAS, GNAI1, DRD5* |
| mmu04060:Cytokine-cytokine receptor interaction | 10.45 | 2.26 | *CXCL10, CCL6, CCR9, CXCR5, XCL1, PPBP, IL7R* |
| mmu04924:Renin secretion | 8.96 | 3.97 | *EDNRA, GNAQ, GNAS, ADRB1, ADRB2, GNAI1* |
| mmu04970:Salivary secretion | 8.96 | 3.80 | *CHRM3, GNAQ, GNAS, ADRB1, ADRB2, VAMP2* |
| mmu05032:Morphine addiction | 8.96 | 3.41 | *GNGT1, GNG5, GNB2, GNAS, ARRB2, GNAI1* |
| mmu04713:Circadian entrainment | 8.96 | 3.31 | *GNGT1, GNG5, GNB2, GNAQ, GNAS, GNAI1* |
| mmu04723:Retrograde endocannabinoid signaling | 8.96 | 3.21 | *GNGT1, GNG5, CNR1, GNB2, GNAQ, GNAI1* |
| mmu04724:Glutamatergic synapse | 8.96 | 2.99 | *GNGT1, GNG5, GNB2, GNAQ, GNAS, GNAI1* |
| mmu04120:Ubiquitin mediated proteolysis | 8.96 | 2.59 | *SOCS3, CUL7, UBA7, MGRN1, CDC27, ANAPC5* |
| mmu04022:cGMP-PKG signaling pathway | 8.96 | 2.33 | *EDNRA, GNA11, GNAQ, ADRB1, ADRB2, GNAI1* |
| mmu04923:Regulation of lipolysis in adipocytes | 7.46 | 3.25 | *GNAS, ADRB1, PIK3R2, ADRB2, GNAI1* |
| mmu04540:Gap junction | 7.46 | 2.58 | *GNA11, GNAQ, GNAS, ADRB1, GNAI1* |
| mmu04915:Estrogen signaling pathway | 7.46 | 2.38 | *HSPA8, GNAQ, GNAS, PIK3R2, GNAI1* |
| mmu05142:Chagas disease (American trypanosomiasis) | 7.46 | 2.30 | *GNA11, GNAQ, GNAS, PIK3R2, GNAI1* |
| mmu05146:Amoebiasis | 7.46 | 2.11 | *RAB5B, GNA11, GNAQ, GNAS, PIK3R2* |
| mmu04261:Adrenergic signaling in cardiomyocytes | 7.46 | 1.82 | *GNAQ, GNAS, ADRB1, ADRB2, GNAI1* |
| mmu05034:Alcoholism | 7.46 | 1.33 | *GNGT1, GNG5, GNB2, GNAS, GNAI1* |
| mmu04962:Vasopressin-regulated water reabsorption | 5.97 | 2.53 | *RAB5B, AVPR2, GNAS, VAMP2* |
| mmu04730:Long-term depression | 5.97 | 2.10 | *GNA11, GNAQ, GNAS, GNAI1* |
| mmu04971:Gastric acid secretion | 5.97 | 1.90 | *CHRM3, GNAQ, GNAS, GNAI1* |
| mmu04914:Progesterone-mediated oocyte maturation | 5.97 | 1.68 | *CDC27, ANAPC5, PIK3R2, GNAI1* |
| mmu04727:GABAergic synapse | 5.97 | 1.68 | *GNGT1, GNG5, GNB2, GNAI1* |
| mmu04916:Melanogenesis | 5.97 | 1.54 | *GNAQ, WNT5A, GNAS, GNAI1* |
| Module 2 |  |  |  |
| mmu03040:Spliceosome | 21.05 | 10.26 | *PRPF4, XAB2, PPIL1, HNRNPA3, SF3B2, PRPF6, PCBP1, SNRPG, PPIH, SNRPB2, BCAS2, SNRPA* |
| mmu04610:Complement and coagulation cascades | 7.02 | 2.12 | *SERPINA1B, VWF, SERPINE1, CD55* |
| mmu05215:Prostate cancer | 7.02 | 1.95 | *CDKN1B, CREB1, FOXO1, MTOR* |
| mmu04962:Vasopressin-regulated water reabsorption | 5.26 | 1.66 | *CREB1, DCTN1, DYNLL2* |
| Module 3 |  |  |  |
| mmu05166:HTLV-I infection | 16.28 | 3.00 | *EGR1, CD40, CDKN1A, CD3E, IL2RG, H2-AB1, ATF4* |
| mmu05145:Toxoplasmosis | 13.95 | 4.12 | *CD40, CASP3, IRGM1, MAPK14, HSPA1B, H2-AB1* |
| mmu05164:Influenza A | 13.95 | 3.13 | *TLR7, MAPK14, ACTB, IRF9, HSPA1B, H2-AB1* |
| mmu04010:MAPK signaling pathway | 13.95 | 2.40 | *NTRK2, DUSP1, CASP3, MAPK14, HSPA1B, ATF4* |
| mmu04668:TNF signaling pathway | 11.63 | 2.94 | *CEBPB, CASP3, MAPK14, JUNB, ATF4* |
| mmu04380:Osteoclast differentiation | 11.63 | 2.71 | *FOSB, MAPK14, JUNB, IRF9, FOSL2* |
| mmu05162:Measles | 11.63 | 2.59 | *TLR7, CD3E, IL2RG, IRF9, HSPA1B* |
| mmu05030:Cocaine addiction | 9.30 | 2.94 | *DLG4, FOSB, GRIN2B, ATF4* |
| mmu05416:Viral myocarditis | 9.30 | 2.34 | *CD40, CASP3, ACTB, H2-AB1* |
| mmu04068:FoxO signaling pathway | 9.30 | 1.72 | *CDKN1A, BCL2L11, PTEN, MAPK14* |
| mmu05161:Hepatitis B | 9.30 | 1.62 | *CDKN1A, CASP3, PTEN, ATF4* |
| mmu04390:Hippo signaling pathway | 9.30 | 1.58 | *DLG4, CSNK1D, AREG, ACTB* |
| mmu05152:Tuberculosis | 9.30 | 1.41 | *CEBPB, CASP3, MAPK14, H2-AB1* |
| mmu05340:Primary immunodeficiency | 6.98 | 2.04 | *CD40, CD3E, IL2RG* |
| mmu05014:Amyotrophic lateral sclerosis (ALS) | 6.98 | 1.70 | *CASP3, MAPK14, GRIN2B* |
| mmu05134:Legionellosis | 6.98 | 1.61 | *CASP3, EEF1A2, HSPA1B* |
| mmu05031:Amphetamine addiction | 6.98 | 1.48 | *FOSB, GRIN2B, ATF4* |
| mmu04115:p53 signaling pathway | 6.98 | 1.48 | *CDKN1A, CASP3, PTEN* |
| Module 4 |  |  |  |
| mmu04146:Peroxisome | 71.43 | 6.73 | *GNPAT, ACOT8, SCP2, EHHADH, CRAT* |
| mmu01100:Metabolic pathways | 71.43 | 2.08 | *ACOT8, SCP2, EHHADH, ACOT4, ACOT3* |
| mmu00120:Primary bile acid biosynthesis | 28.57 | 1.91 | *ACOT8, SCP2* |
| mmu00062:Fatty acid elongation | 28.57 | 1.70 | *ACOT4, ACOT3* |
| mmu01040:Biosynthesis of unsaturated fatty acids | 28.57 | 1.68 | *ACOT4, ACOT3* |
| Module 5 |  |  |  |
| mmu04921:Oxytocin signaling pathway | 13.21 | 4.09 | *PPP3R1, CD38, FOS, PRKACA, EEF2, PPP1CA, ACTG1* |
| mmu05166:HTLV-I infection | 13.21 | 2.67 | *PPP3R1, EGR2, IL2RB, FOS, PRKACA, ATF3, JAK1* |
| mmu04114:Oocyte meiosis | 11.32 | 3.69 | *PPP3R1, PPP2R5B, PPP2R5A, PRKACA, YWHAG, PPP1CA* |
| mmu04151:PI3K-Akt signaling pathway | 11.32 | 1.55 | *IL2RB, PPP2R5B, PPP2R5A, TLR4, YWHAG, JAK1* |
| mmu05031:Amphetamine addiction | 9.43 | 3.51 | *PPP3R1, ARC, FOS, PRKACA, PPP1CA* |
| mmu04728:Dopaminergic synapse | 9.43 | 2.39 | *PPP2R5B, PPP2R5A, FOS, PRKACA, PPP1CA* |
| mmu03013:RNA transport | 9.43 | 2.02 | *POM121, EIF3E, EIF2S3X, EIF3C, EIF4G2* |
| mmu05205:Proteoglycans in cancer | 9.43 | 1.76 | *PTPN11, PRKACA, TLR4, PPP1CA, ACTG1* |
| mmu05323:Rheumatoid arthritis | 7.55 | 2.12 | *MMP3, CTLA4, FOS, TLR4* |
| mmu05162:Measles | 7.55 | 1.54 | *IL2RB, TLR4, SLAMF1, JAK1* |
| mmu04261:Adrenergic signaling in cardiomyocytes | 7.55 | 1.49 | *PPP2R5B, PPP2R5A, PRKACA, PPP1CA* |
| mmu05161:Hepatitis B | 7.55 | 1.46 | *EGR2, FOS, TLR4, JAK1* |
| mmu05140:Leishmaniasis | 5.66 | 1.40 | *FOS, TLR4, JAK1* |
| mmu04720:Long-term potentiation | 5.66 | 1.38 | *PPP3R1, PRKACA, PPP1CA* |
| Module 6 |  |  |  |
| mmu01100:Metabolic pathways | 50 | 2.36 | *CYP2C37, CYP2C68, PLA2G2D, CYP3A44, PLA2G6, CYP2C39, CYP4F13* |
| mmu00591:Linoleic acid metabolism | 42.86 | 8.37 | *CYP2C37, CYP2C68, PLA2G2D, CYP3A44, PLA2G6, CYP2C39* |
| mmu00590:Arachidonic acid metabolism | 42.86 | 7.09 | *CYP2C37, CYP2C68, PLA2G2D, PLA2G6, CYP2C39, CYP4F13* |
| mmu00140:Steroid hormone biosynthesis | 35.71 | 5.324 | *CYP2C37, SULT2B1, CYP2C68, CYP3A44, CYP2C39* |
| mmu00830:Retinol metabolism | 28.57 | 3.636 | *CYP2C37, CYP2C68, CYP3A44, CYP2C39* |
| mmu05204:Chemical carcinogenesis | 28.57 | 3.59 | *CYP2C37, CYP2C68, CYP3A44, CYP2C39* |
| mmu04750:Inflammatory mediator regulation of TRP channels | 28.57 | 3.19 | *CYP2C37, CYP2C68, PLA2G6, CYP2C39* |
| mmu04726:Serotonergic synapse | 21.43 | 1.84 | *CYP2C37, CYP2C68, CYP2C39* |
| mmu00592:alpha-Linolenic acid metabolism | 14.29 | 1.45 | *PLA2G2D, PLA2G6* |
| Module 7 |  |  |  |
| mmu03013:RNA transport | 21.43 | 2.33 | *EIF4A1, EIF4EBP2, EIF4G1* |
| ATS use Cluster 8 |  |  |  |
| mmu04068:FoxO signaling pathway | 25.00 | 3.98 | *CCNB1, STAT3, IRS2, SLC2A4, KLF2* |
| mmu05203:Viral carcinogenesis | 25.00 | 3.08 | *GTF2A1, CCND3, STAT3, TBPL1, GTF2H4* |
| mmu03022:Basal transcription factors | 20.00 | 4.12 | *GTF2A1, ERCC2, TBPL1, GTF2H4* |
| mmu05206:MicroRNAs in cancer | 20.00 | 1.82 | *STAT3, IRS2, VIM, CD44* |
| mmu04920:Adipocytokine signaling pathway | 15.00 | 2.08 | *STAT3, IRS2, SLC2A4* |
| mmu04640:Hematopoietic cell lineage | 15.00 | 1.94 | *CD24A, CD34, CD44* |
| mmu04931:Insulin resistance | 15.00 | 1.72 | *STAT3, IRS2, SLC2A4* |
| mmu04152:AMPK signaling pathway | 15.00 | 1.60 | *RAB2A, IRS2, SLC2A4* |
| mmu05169:Epstein-Barr virus infection | 15.00 | 1.55 | *STAT3, VIM, CD44* |
| Opioid treatment |  |  |  |
| Module 1 |  |  |  |
| mmu05205:Proteoglycans in cancer | 38.46 | 4.27 | *TRP53, IGF2, KDR, EGFR, CD44* |
| mmu04151:PI3K-Akt signaling pathway | 38.46 | 3.35 | *INS1, TRP53, SPP1, KDR, EGFR* |
| mmu05215:Prostate cancer | 23.08 | 2.35 | *INS1, TRP53, EGFR* |
| mmu04510:Focal adhesion | 23.08 | 1.64 | *SPP1, KDR, EGFR* |
| mmu04015:Rap1 signaling pathway | 23.08 | 1.61 | *INS1, KDR, EGFR* |
| mmu04014:Ras signaling pathway | 23.08 | 1.56 | *INS1, KDR, EGFR* |
| mmu05206:MicroRNAs in cancer | 23.08 | 1.41 | *TRP53, EGFR, CD44* |
| mmu05219:Bladder cancer | 15.38 | 1.33 | *TRP53, EGFR* |
| Module 2 |  |  |  |
| mmu05200:Pathways in cancer | 36.84 | 4.90 | *FZD1, GNGT1, FZD2, LPAR3, WNT9A, FOS, WNT3* |
| mmu04550:Signaling pathways regulating pluripotency of stem cells | 26.32 | 4.36 | *FZD1, FZD2, PAX6, WNT9A, WNT3* |
| mmu04310:Wnt signaling pathway | 26.32 | 4.32 | *VANGL1, FZD1, FZD2, WNT9A, WNT3* |
| mmu04390:Hippo signaling pathway | 26.32 | 4.20 | *FZD1, FZD2, WNT9A, AFP, WNT3* |
| mmu05166:HTLV-I infection | 26.32 | 3.19 | *FZD1, FZD2, WNT9A, FOS, WNT3* |
| mmu05217:Basal cell carcinoma | 21.05 | 4.16 | *FZD1, FZD2, WNT9A, WNT3* |
| mmu04916:Melanogenesis | 21.05 | 3.38 | *FZD1, FZD2, WNT9A, WNT3* |
| mmu05205:Proteoglycans in cancer | 21.05 | 2.48 | *FZD1, FZD2, WNT9A, WNT3* |

Table S4 Top 20 hub genes ranked by MCC socre by CytoHubba Plugin with MCC algorithms

| Rank | Opioid treatment DEGs | | ATS treatment DEGs | | CDEGs | |
| --- | --- | --- | --- | --- | --- | --- |
|  | Gene symbol | MCC Score | Gene symbol | MCC Score | Gene symbol | MCC Score |
| 1 | *Spp1* | 3853 | *Adrb2* | 4.19×10^13^ | *Fos* | 13 |
| 2 | *Trp53* | 3355 | *Avpr2* | 4.19×10^13^ | *Dusp1* | 10 |
| 3 | *Egfr* | 3241 | *Il7r* | 4.18×10^13^ | *Sgk1* | 8 |
| 4 | *Cd44* | 3081 | *Tfrc* | 4.18×10^13^ | *Nr4a2* | 5 |
| 5 | *Igf2* | 3035 | *Syt1* | 4.18×10^13^ | *Ddit4* | 4 |
| 6 | *Afp* | 2185 | *Vamp2* | 4.18×10^13^ | *Hspa1b* | 4 |
| 7 | *Ins1* | 1631 | *Lrp2* | 4.18×10^13^ | *Npas4* | 4 |
| 8 | *Sox9* | 1623 | *Sh3gl1* | 4.18×10^13^ | *Cdkn1a* | 3 |
| 9 | *Kdr* | 1487 | *Dnajc6* | 4.18×10^13^ | *Cd44* | 3 |
| 10 | *F5* | 727 | *Wnt5a* | 4.18×10^13^ | *Rasd1* | 2 |
| 11 | *Serpinc1* | 726 | *Wasl* | 4.18×10^13^ | *Tsc22d3* | 2 |
| 12 | *Dmp1* | 722 | *Arpc4* | 4.18×10^13^ | *Syk* | 2 |
| 13 | *Prss23* | 721 | *Arrb2* | 4.18×10^13^ | *Arc* | 2 |
| 14 | *Ktn1* | 720 | *Sh3gl2* | 4.18×10^13^ | *Gngt1* | 2 |
| 15 | *Fos* | 337 | *Sgip1* | 4.18×10^13^ | *Spam1* | 1 |
| 16 | *Calb2* | 240 | *Itsn2* | 4.18×10^13^ | *Ern1* | 1 |
| 17 | *Pax6* | 207 | *Hspa8* | 2.09×10^13^ | *Cpsf3* | 1 |
| 18 | *Olig2* | 189 | *Rab5b* | 2.09×10^13^ | *Ssbp1* | 1 |
| 19 | *Ascl1* | 186 | *Gnb2* | 1.40×10^12^ | *Kcnj2* | 1 |
| 20 | *Slc17a7* | 156 | *Gngt1* | 1.40×10^12^ | *Txnip* | 1 |

Table S5 The relative mRNA levels of hub genes in BV2 cells treated with 1000 μM of METH and 200 μM of Heroin (Mean ±SD)

| Gene | METH | Heroin | Control | *P*^1^ | *P*^2^ |
| --- | --- | --- | --- | --- | --- |
| *CD11b* | 5.21±2.27 | 14.87±5.96 | 1.02±0.17 | 0.004* | 0.004* |
| *TNF-a* | 2.85±1.24 | 6.39±1.87 | 1.04±0.31 | 0.016* | ＜0.001 |
| *IL-6* | 4.87±2.85 | 4.65±2.11 | 1.15±0.54 | 0.028* | 0.003 |
| *Fos* | 6.35±4.42 | 24.83±9.74 | 1.08±0.49 | 0.037* | 0.004* |
| *Dusp1* | 1.91±0.68 | 4.51±0.97 | 1.06±0.40 | ＜0.001 | ＜0.001 |
| *Sgk1* | 9.98±6.66 | 17.45±8.49 | 1.01±0.35 | 0.004* | 0.004* |
| *Nr4a2* | 2.10±0.92 | 9.15±3.56 | 1.00±0.26 | 0.018 | 0.004* |
| *Ddit4* | 19.34±9.54 | 36.01±4.99 | 1.71±0.88 | 0.004* | 0.004* |
| *Cdkn1a* | 2.93±0.81 | 16.41±6.29 | 0.96±0.12 | 0.004* | 0.004* |
| *PI3K* | 1.92±0.69 | 10.05±3.83 | 1.05±0.43 | 0.027 | 0.004* |
| *AKT* | 3.41±1.52 | 23.48±9.39 | 1.35±0.73 | 0.014 | ＜0.001 |

*P*^1^, METH *vs.*Controls; *P*^2^,Heroin *vs.*Controls. ^*^ Kruskal-Wallis Test
